# Novel mesostructured inclusions in the epidermal lining of *Artemia franciscana* ovisacs show optical activity

**DOI:** 10.7717/peerj.3923

**Published:** 2017-10-27

**Authors:** Elena Hollergschwandtner, Thomas Schwaha, Josef Neumüller, Ulrich Kaindl, Daniela Gruber, Margret Eckhard, Michael Stöger-Pollach, Siegfried Reipert

**Affiliations:** 1 Core Facility Cell Imaging and Ultrastructure Research, University of Vienna, Vienna, Austria; 2 Department of Integrative Zoology, University of Vienna, Vienna, Austria; 3 Center of Anatomy and Cell Biology, Medical University of Vienna, Vienna, Austria; 4 University Service Center for TEM (USTEM), Vienna University of Technology, Vienna, Austria

**Keywords:** *Artemia franciscana*, Brine shrimps, Confocal reflection microscopy, Rapid freeze substitution, Ovisac, Reproduction, Cellular inclusions, Mesocrystals, Transmission electron microscopy

## Abstract

**Background:**

Biomineralization, e.g., in sea urchins or mollusks, includes the assembly of mesoscopic superstructures from inorganic crystalline components and biopolymers. The resulting mesocrystals inspire biophysicists and material scientists alike, because of their extraordinary physical properties. Current efforts to replicate mesocrystal synthesis *in vitro* require understanding the principles of their self-assembly *in vivo*. One question, not addressed so far, is whether intracellular crystals of proteins can assemble with biopolymers into functional mesocrystal-like structures. During our electron microscopy studies into *Artemia franciscana* (Crustacea: Branchiopoda), we found initial evidence of such proteinaceous mesostructures.

**Results:**

EM preparations with high-pressure freezing and accelerated freeze substitution revealed an extraordinary intracellular source of mesostructured inclusions in both the cyto-and nucleoplasm of the epidermal lining of ovisacs of *A. franciscana*. Confocal reflection microscopy not only confirmed our finding; it also revealed reflective, light dispersing activity of these flake-like structures, their positioning and orientation with respect to the ovisac inside. Both the striation of alternating electron dense and electron-lucent components and the sharp edges of the flakes indicate self-assembly of material of yet unknown origin under supposed participation of crystallization. However, selected area electron diffraction could not verify the status of crystallization. Energy dispersive X-ray analysis measured a marked increase in nitrogen within the flake-like inclusion, and the almost complete absence of elements that are typically involved in inorganic crystallization. This rise in nitrogen could possibility be related to higher package density of proteins, achieved by mesostructure assembly.

**Conclusions:**

The ovisac lining of *A. franciscana* is endowed with numerous mesostructured inclusions that have not been previously reported. We hypothesize that their self-assembly was from proteinaceous polycrystalline units and carbohydrates. These mesostructured flakes displayed active optical properties, as an umbrella-like, reflective cover of the ovisac, which suggests a functional role in the reproduction of *A. franciscana*. In turn, studies into ovisac mesostructured inclusions could help to optimizing rearing *Artemia* as feed for fish farming. We propose *Artemia* ovisacs as an *in vivo* model system for studying mesostructure formation.

## Introduction

In recent years, brine shrimps *Artemia franciscana* (Crustacea: Branchiopoda) have attracted much interest of both research and fishery. The former explores the molecular basis for their exceptional stress tolerance, the reproduction pathways, and the role of nutrients and pathogens for their life cycle (McRae, 2003; Gajardo & Beardmore, 2012). The latter uses unique possibilities for industrial breeding of these crustaceans as a valuable fish food supply.

**Figure 1:**
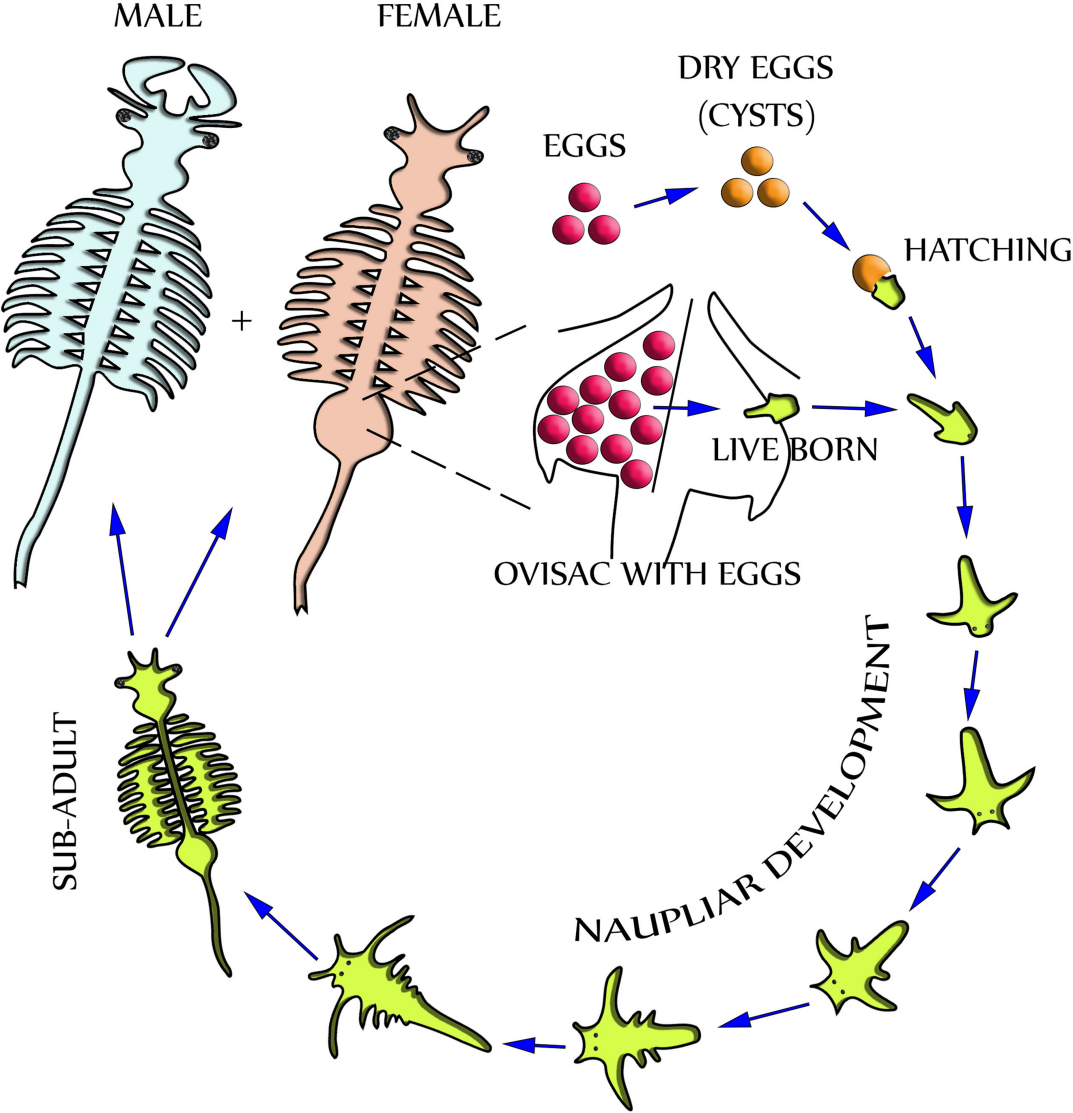
Life cycle of *Artemia Franciscana.* Two distinctly different paths of reproduction are possible: Under favorable environmental conditions embryos develop directly inside the ovisac, and nauplii are released from there (ovoviviparity). Under harsh environmental conditions the ovisac produces diapausing eggs that dry out after their release (oviparity). Once the cysts are rehydrated, larvae (nauplii) will hatch. Notably, light has been identified as a factor for inducing hatching (Sorgeloos, 1973). The development of nauplii into sub-adults occurs within 1–3 weeks. Adult females release oocytes from their two oviducts into the ovisac where they become fertilized by males.

Dependent upon environmental and seasonal factors, the reproduction of *A. franciscana*, illustrated in Fig. 1, may follow either of two different paths: Under less favorable conditions, reproduction is realized by oviparous release of eggs (cysts) at a stage of diapause. Alternatively, under more favorable conditions, reproduction is by ovoviviparous development of eggs inside the ovisac (uterus) and instant hatching of nauplii (Criel & MacRae, 2002). The ovisac of female *Artemia* facilitates major processes of the reproduction process: Once the ovaries have released oocytes into two lateral pouches (oviducts) they undergo vitellogenesis. During mating the males deposit sperm inside ovisacs, where it matures and initiates embryogenesis by fertilization of the eggs. Notably, some strains of *A. franciscana* undergo embryogenesis without fertilization (known as reproduction by parthenogenesis).

Different microscopy methods have been used to study *Artemia* life cycle. Stereomicroscopy is routinely applied to evaluate the reproduction process in the ovisac of *A. franciscana*, and to distinguish between stages of oogenesis, egg- and cyst maturation, and activity of the shell glands (Liang & MacRae, 1999; Dai et al., 2011). Detailed characterization of the reproductive processes requires the use of transmission electron microscopy (TEM). Previous comprehensive TEM studies of chemically fixed and embedded samples were focused at developmental processes inside the ovisac (Criel, 1980a; Criel, 1980b). However, they did not report specific observations concerning the cuticle and subjacent epithelial cell layer of the ovisac itself. Studies of the formation of epidermis were performed at the larval stage of *A. franciscana*, but this local and time-dependent process was not followed up to maturation of the animal (Wolf et al., 1989; Freeman, 1986; Freeman & Chronister, 1989). TEM studies of adult epidermis aimed specifically to understand molting of the cuticle, but the ovisac was never chosen for these investigations (Criel & Walgraeve, 1989). Our report, therefore, complements previous studies, which excluded the ovisac lining from TEM investigation. Moreover, we note that previous TEM studies of *A. franciscana* relied on conventional chemical sample preparation at room temperature. This opens ample opportunities for further improvement of sample preparation by cryotechniques that are based on instant freezing. Our EM facility included *A. franciscana* as model organisms in tests of an agitation module for accelerated freeze substitution (FS) (Goldammer et al., 2016). The findings of cellular inclusions within high-pressure frozen epidermal cells of the ovisac, described subsequently, underline the importance of this low-temperature dehydration- and fixation technique for proper preservation of cellular structures for TEM.

Our initial observation of cellular inclusions in cryopreserved epidermal cells inspired expanding our studies to a more comprehensive characterization of the ovisac lining by different methods including Confocal Reflection Microscopy (CRM) (for review: Paddock, 2002), electron tomography, and selected area electron diffraction (SAED). Taken together, these studies revealed aspects of the mesostructured nature of these ovisac inclusions. In addition, they revealed the organization of these entities as reflective, light-dispersing ‘umbrella’ of the ovisac. We discuss our findings in the framework of *A. franciscana* reproduction, and we highlight both common ground and differences with respect to mesocrystalline superstructures known from biomineralization (Cölfen & Antonietti, 2005; Bergström et al., 2015).

## Materials & Methods

### Culturing *Artemia Franciscana*

Dried cysts of *A. franciscana* strains, from Vinh Chau (Vietnam), Great Salt Lake, San Francisco Bay and TUZ (Turkey) were provided by Prof. van Stappen, Laboratory of Aquaculture & Artemia Reference Center, University of Gent. The larvae were reared under laboratory conditions to the adult stage as follows: 350 g of sea salt for aqua culturing ‘Reef crystals’ (Aquarium Systems, Grab, Switzerland) were dissolved in 10 l double-distilled water and applied for cultivation. The salinity, between 35 to 40 ppt, was controlled with a refractometer (Aqua Medic GmbH, Bissendorf, Germany). Hatching of nauplii from the cysts occurred in Duran^®^ vessels, 6 cm high and 10 cm in diameter, filled with 200 ml artificial seawater. After two days, nauplii were selected into culture medium bottles with a capacity of 500 ml seawater. The number of individuals was limited to 15 to 20 nauplii per bottle. LED arrays (AuraLum No. S3030-18 W, 2,800–3,200 K, white light; 2 × 40 cm × 40 cm) were placed at a distance of 30 cm from the culture bottles for illumination (16 h/day). The water temperature varied between 22 to 28 °C depending on the seasons. For feeding, algae of the two strains, *Rhodomonas* sp. and *Tisochrysis lutea*, strain 927/14 (Collection of Algae and Protozoa) (courtesy of Dr. Daniel Abed-Navandi, Haus des Meeres Vienna) were added to the culture.

### Stereomicroscopy

Freshly dissected ovisacs of *A. franciscana* were analyzed and documented with a Nikon SMZ25 stereomicroscope (Nikon, Tokyo, Japan) equipped with a Nikon DS-Ri1 U3 microscope camera. Specimens were obliquely illuminated with LED spots. Images of *z*-stacks were recorded and merged with the Nikon NIS-Elements BR software. The position of the LED spots was adjusted to find out the optimal reflective conditions for the mesostructured inclusions covering the ovisac. Movies of *A. francisana* were recorded with a Nikon J1 system camera mounted on a Nikon SMZ25 stereomicroscope using incident-oblique or transmitted light.

### Polarisation microscopy

Dissected linings of the ovisac were mounted in 0.1 M phosphate buffered saline (PBS), pH 7.4, and analyzed under cross-polarized light in a Nikon Eclipse E800 (Nikon, Tokyo, Japan) light microscope equipped with a Nikon DsFi2-U3 microscope camera.

### Confocal laser scanning microscopy (CLSM)

For analyzing cuticular autofluorescence, ovisacs of *A. franciscana* were cut off and mounted on microscopic slides in ≥99,5% glycerol Rotipuran^®^ (Roth GmbH, Karlsruhe, Germany). Autofluorescence of the specimens was recorded using Leica SP5 II confocal laser scanning microscope with LAS-AF software (Leica Microsystems, Wetzlar, Germany). Scans were either taken with a 20× or a 63× objective and confocal *z*-stacks were recorded at step sizes of 1 and 0.4 µm, respectively. The pinhole was set to one airy unit (AU) for low magnification and to 0.5 AU for high magnification. Autofluorescence resulting from excitation with wavelengths of 633 nm, 561 nm, 488 nm and 405 nm was recorded as previously described for other arthropods (Michels, 2007). The emitted fluorescence light was recorded with a photomultiplier tube or a Leica HyD detector at longer wavelength, above the respective excitation.

For visualization of the reflection of cellular inclusions in the epidermal lining, ovisacs were transferred directly to 0.1 M PBS, pH 7.4, since mounting in glycerol was unfavorable for reflection. Reflection images were recorded for three laser wave lines, at 633 nm, 561 nm and 488 nm. Windows for detection of the reflection signals were set within the narrow boundaries of the respective excitation spectra (Michels, 2007). Reflection images were displayed as single z-slices and as maximum intensity projections of parts or the whole image stack using the LAS-AF software or FIJI (Schindelin et al., 2012).

### Conventional chemical preparation of TEM samples at room temperature

Adult females, with their eggs assembled in a single mass inside of the uterus, were transferred into embryo dishes on ice. Once their movement was reduced they were decapitated with a microscissor and the ovisac was dissected. This procedure took less than 2 min for each animal. Dissected ovisacs, were immersed in 3 ml fixative consisting of 4% paraformaldehyde or 2.5% glutaraldehyde dissolved in PHEM buffer (90 mM PIPES, 37.5 mM HEPES, 15 mM EGTA and 3 mM MgCl_2_; pH 7.4 with 5% sucrose added), as described recently by Montanaro, Gruber & Leisch (2016) for improvement in preservation of marine invertebrates. The chemical fixation of the samples in glass vials (V-vials; EMS, Hatfield, PA, USA) was supported by microwave (MW) exposure for 30 min at 200 W in a temperature-controlled laboratory MW oven, PELCO BioWave^®^ Pro (Ted Pella, Inc, Redding, CA, USA) equipped with a ‘steady state’ tray for more even distribution of the MWs. To avoid overheating of samples, the temperature was limited to 36 °C. After postfixation at 4 °C overnight, samples were washed in PHEM buffer, and subsequently immersed in 1% osmium tetroxide (OsO_4_) in double-distilled water for 1 h. Dehydration by ethanol series (30%, 50%, 70% and 95% ethanol for 10 min each and two times 100% ethanol for 10 min each) was followed by immersion in propylene oxide. The infiltration with low-viscosity epoxy resin (Agar Scientific, Stansted, UK) was performed step-wise in solvent/ resin mixtures. Polymerization in freshly prepared, pure resin was accomplished over at least two days in an oven at 60 °C.

### High-pressure freezing and freeze substitution (FS) accelerated by agitation

Ovisacs, dissected as described above, were transferred into carriers type A (6 mm diameter; 200 µm depth) and covered with the flat surface of carriers type B (LEICA Microsystems, Wien, Austria). Notably, the carriers were coated with 1-hexadecene prior to use, and the inside volume of carrier A was filled up with 10% bovine serum albumin (BSA). The carrier sandwich was inserted into the middle plate of a sample cartridge and high-pressure frozen (HP-frozen) with the freezer HPM100 (LEICA Microsystems, Austria). FS was performed in an automatic freeze substitution unit AFS2 (Leica Microsystems, Austria) equipped with a self-made agitation module described elsewhere (Goldammer et al., 2016). Carriers containing the HP-frozen samples were placed onto the frozen substitution medium (1% OsO_4_ in acetone) in Sarstedt tubes. For Selected area electron diffraction (SAED), ovisacs of the TUZ strain from Turkey were also freeze-substituted in acetone in absence of OsO_4_. FS took place under agitation at −85 °C, initially for 3 h, but mostly for 10 h overnight. It was followed by warming up to room temperature and embedding in low viscosity epoxy resin (Agar Scientific, Stansted, UK). The temperature/time course was measured and recorded with a K- type thermocouple and USB data logger EL- USB-TC- LCD (Lascar Electronics, Erie, PA, USA) inside a dummy tube filled with acetone.

### EDX

Energy-dispersive X-ray microanalysis (EDX) was performed with an AMETEK EDAX Octane Plus detector attached to a JEOL IT300 scanning electron microscope equipped with a LaB_6_-cathode. Semithin sections of HP- frozen, rapidly freeze-substituted embedding of ovisacs, 2.5 µm in thickness, were placed on silicon wafers, mounted with carbon tabs onto aluminum stubs and coated with carbon by using a Leica EM MED020 vacuum coating system. Areas containing cellular inclusions were identified with SE-and BSE detectors and selected for measurement. Spectra and dot-mappings were recorded at 20 kV, at a working distance of 11 mm and under 35° -positioning of the EDAX detector. The dead-time of the detector was set between 22 and 25 s, and its lifetime at 30 s. Data collection, automated background subtraction and analysis were performed using the EDAX TEAM Software Version (V4.20) AMETEK GmbH, EDAX Division.

### Selected area electron diffraction

Selected area electron diffraction (SAED) and high-resolution TEM imaging at 200 kV was applied to 60 nm epoxy resin sections of ovisacs that were processed by HP freezing and rapid FS. For SAED at 200 kV a 10 µm-sized aperture is placed into the first image of the objective lens and the focal plane is projected to the camera system of a TECNAI F20 transmission electron microscope (FEI, Eindhoven, The Netherlands). Due to elastic scattering, some electron trajectories are deflected off the optical axes, thus forming the diffraction patterns. In the case of amorphous structures, diffuse rings show the medium atomic distance.

### Electron tomography

Electron tomography of epoxy resin sections of HP frozen, rapidly freeze-substituted ovisacs of *A. franciscana* was performed using a Tecnai-20 electron microscope at 200 kV equipped with an eucentric goniometer and a single high-tilt holder (FEI, Eindhoven, The Netherlands) Tilt series of digital images within an angular range of 65° to +65° and a tilt increment of 1° were recorded with the help of the Xplore 3D software (FEI company) using a Eagles 4K-CCD camera (FEI Company; chip size: 4.096 × 4.096 pixels). In order to reconstruct the volume of the 200–300 nm thick sections into virtual slices (thickness 0.39–0.46 nm corresponding to a microscope magnification of 29.000× or 25.000× respectively), we used the IMOD software (Boulder Laboratory for 3D Electron Microscopy of Cells, University of Colorado, USA). For 3D-modeling, the structures of interest in each slice were traced with colored contours that were merged in the *Z*-axis with the help of the Amira 5.3 software (Mercury Computer Systems, Merignac, Cedex, France).

## Results

### Stereomicroscopy: variable observation of ovisac glitter

**Figure 2:**
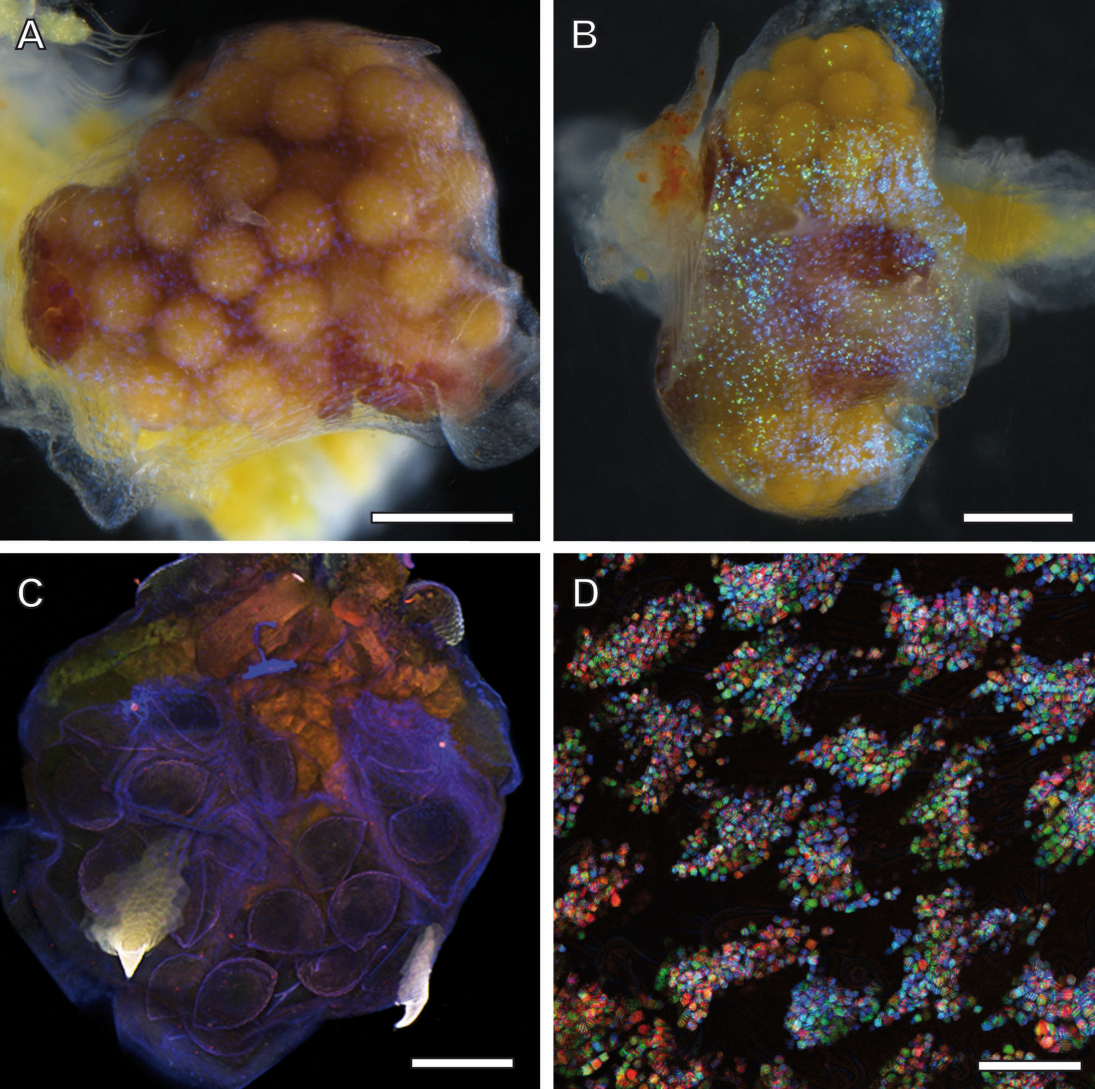
Ovisac of *A. franciscana* visualized by using light microscopy. Depending upon the orientation of the incident illumination, the ovisac lining displays a glitter that is either inconspicuous (A) or very evident (B) under the stereomicroscope. For (B), the LED illumination source was oriented in favor of reflection caused by glittering flakes. (C) Autofluorescence microscopy of maximum intensity projections of CLSM-stack images. The ovisac lining shows variation of the autofluorescence over the surface area, especially in the region of the two characteristic spines, but no modulation of contrasts that could indicate the presence of glittering, flake-like entities observed in the stereomicroscope. (D) Confocal reflection microscopy. Overlay of three wavelengths images generated from a *z*-stack that covers a large field of view of the ovisac. The *z*-stack contains irregularly shaped inclusions in focus regions, which indicate almost complete covering of the ovisac by multicolored, reflective flake-like structures. Bars in (A–C), 0.5 mm. Bar in (D), 25 µm.

Adult females of *A. franciscana* that are progressed in embryogenesis towards unification of their eggs in a single cluster inside the uterus display a regular pattern of ‘glitter’ in their ovisac lining. Depending upon illumination under the stereo microscope, this glittering was either inconspicuous (Fig. 2A) or very evident (Fig. 2B). Notably, glitter similar to Fig. 2A was recorded previously (e.g., stereomicroscopy images in Soniraj (2002)) but not commented. Our findings in the TEM, described in the following as source of this glitter, initiated and inspired optimization of light microscopy for correlative observations. We discovered that the LED illumination source of the stereomicroscopy must be oriented in favor of reflection by glittering flakes within ovisac epidermal cells. Under this optimized illumination, and adjusted for each sample individually, the stereomicroscope can be used for a rough evaluation of the surface coverage of the ovisacs by reflecting entities.

The glittering of the ovisacs was also observed using live imaging under the stereomicroscope. HD-video analysis enabled us to discriminate between initial vitellogenesis of oocytes in the oviduct and later stages that comprise the release of oocytes in the ovisac, fertilization and egg development. The former was not associated with observations of glittering flakes at the ovisac (see Movie S1). Once egg development inside the ovisac started, the glittering flakes became apparent. (Movie S2, provides an undisturbed view at the ovisac after separation of the copulating male). Under our culture conditions, mating occurred prior to the release of oocytes into the ovisac. Evidence for this observation is provided by Movie S3 which displays a male enclosing a female with its clasping organs. As the absence of eggs in the ovisac coincided with the absence of glittering flakes, we surmised that the glitter was not required for optical triggering of the copulation.

### Confocal microscopy: glitter caused by mesostructured, optical active flakes at the ovisac lining

Since the epidermis of the ovisac is covered by an autofluorescent exoskeleton, we tested whether the cuticular autofluorescence itself or a supposed inherent autofluorescence or reflection could be used for visualization of the glittering flakes in the confocal microscope. As demonstrated in Fig. 2C, the ovisac lining shows a dominant bluish autofluorescence over the surface area, which could be caused by excitation of the elastic protein resilin at a wave length of 405 nm (Michels & Gorb, 2012). Multiple components, likely to include sclerotised chitin, add to the almost white autofluorescence of the two characteristic spines (Asem & Sun, 2016). However, no modulation of contrasts was observed that could indicate the presence of the glittering, flake-like entities seen in the stereomicroscope.

**Figure 3:**
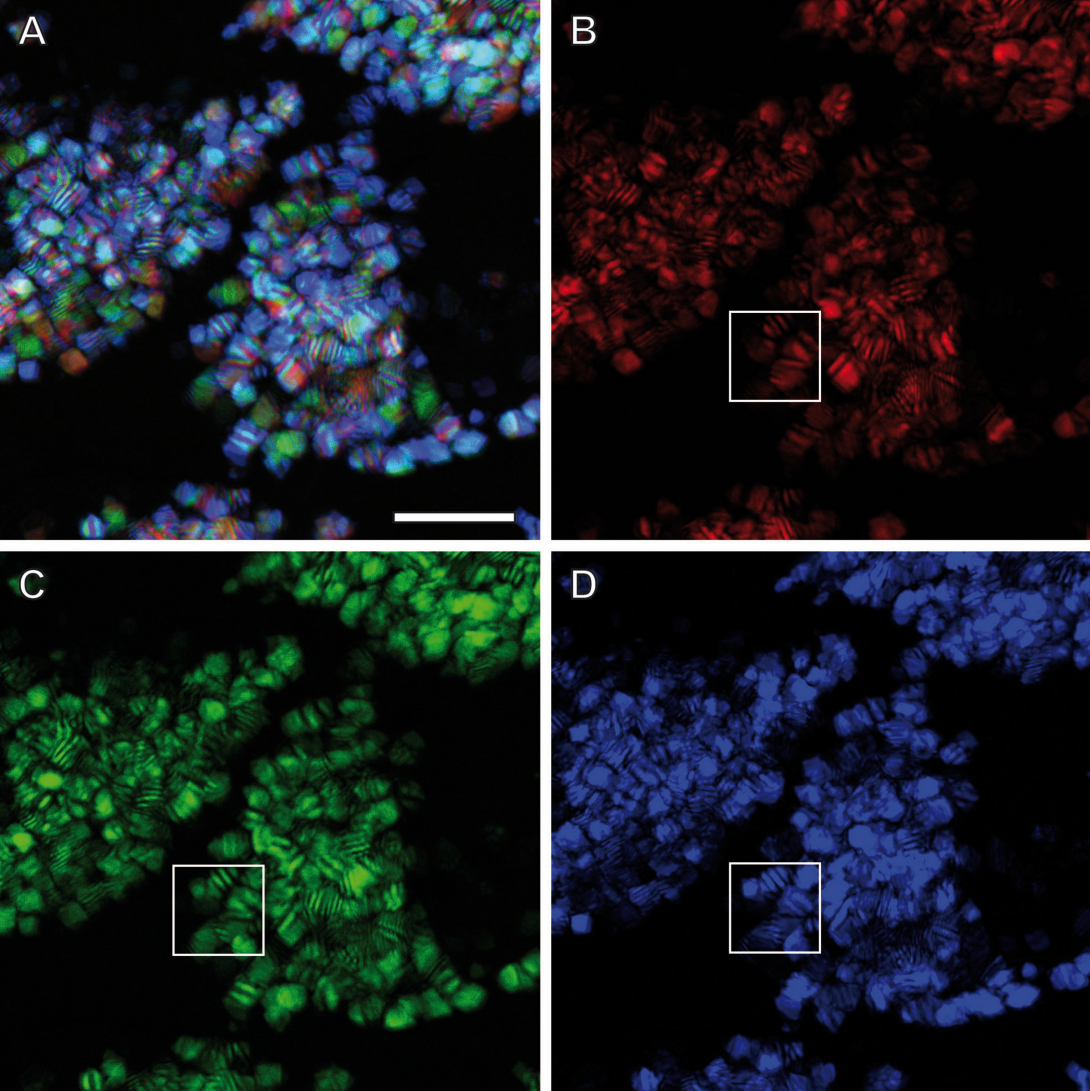
Light dispersion by mesostructured flakes in the ovisac lining of *A. franciscana*. (A) Multicolored confocal reflection microscopy generated from a *z*-stack at high resolution reveals striations within flakes that vary in number and width. (B–D) display the contributions of three individual wavelengths (633 nm, 561 nm and 488 nm, respectively) to the overlay picture (A). The boxed areas mark one and the same region for comparison at different wave lengths. Notably, the image content is wave length-dependent. Bars, 10 µm.

Confocal Reflection Microscopy (CRM) used the reflective optical properties of the glittering elements observed in the stereomicroscope for visualization at higher optical resolution by laser scanning of the ovisac surface with different incident wavelengths (633 nm, 561 nm and 488 nm). Figure 2D displays an overlay of the reflections of all three wavelengths generated from an image *z*-stack that covers a large field of view of the ovisac. Irregularly shaped areas within the focus region of this *z*-stack indicate an almost complete covering of the ovisac by multicolored, reflective flake-like structures, located almost in parallel to the ovisac surface. At higher magnification it became apparent that these flakes possess characteristic mesostructures made of in parallel-running striations (Fig. 3A). If the stack overlay Fig. 3A is separated according to the individual, contributing wavelengths (Figs. 3B–3D), it becomes apparent that one and the same flake is not equally reflective for all three wavelengths. (For comparison the small, boxed area is located at the same position in each of the figures.) As a consequence, each flake of an estimated size of about 25 µm^2^ acts as a dispersive light optical element. Lateral variation in reflection within one and the same flake indicates that the dispersion of light depends on multiple parameters, such as striation patterns, tilt and curvature of the flakes. Visualization of the mesostructures by using a polarization light microscope (Fig. S1) confirmed our expectations that the striated inclusions possess inherent birefringent properties. The images obtained by polarization microscopy, however, lacked the resolution achieved by CRM.

### TEM preparation at room temperature: crystalline cellular inclusions identified but flake-like mesostructures lost

CRM of native samples, in absence of any chemical fixative, exposes reflective mesostructures but it does not answer the question, in which layer of the ovisac lining they are located. Are they attached to the outer surface of the cuticle, are they incorporated into the cuticle or in the epithelial layer of the epidermis? To answer this, we applied TEM to thin sections of epoxy resin-embedded ovisacs.

**Figure 4:**
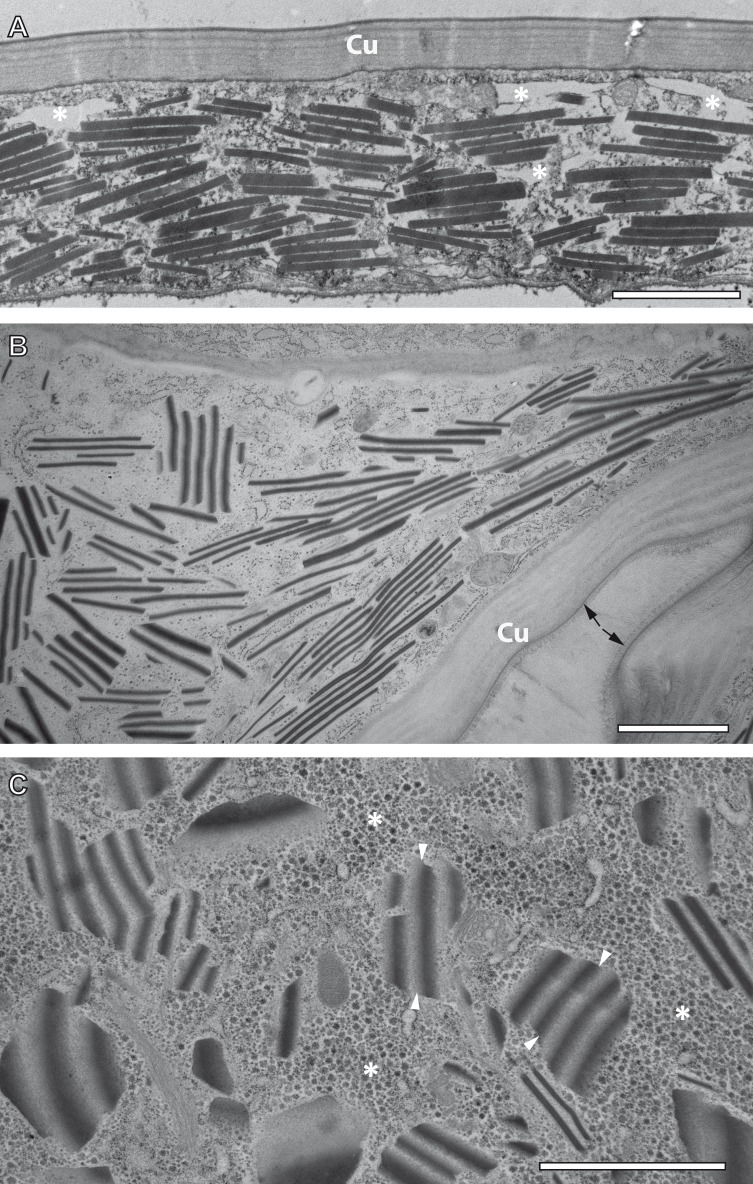
Transmission electron microscopy of mesostructured cytoplasmic inclusion in the epidermal cells of the ovisac lining of *A. franciscana*. (A) Sample chemically fixed and embedded in epoxy resin at room temperature. Rhomboidal inclusions are organized in groups, with individual rhomboids aligned in parallel and proximal to each other. The cytoplasm is severely washed out during sample processing, in particular in areas marked with an asterisk. (B) and (C) Samples high-pressure frozen, followed by freeze substitution and embedding in epoxy resin. (B) shows an epidermal cell detail at low magnification that is well-preserved by cryopreparation, inclusively of a fold in the ovisac lining. Cell organelles, ribosomes and glycogen are apparent. The cuticle (Cu) displays a continuous, brush-like surface layer (arrows), which is not preserved by conventional chemical processing. Given the folding of the epidermis, inclusions are cut at various angles. The impression that groups of aligned electron-dense rhomboids and electron-lucent material in-between are integral parts of common flake-like entities is confirmed at higher magnification in (C). Inclusions cut longitudinally resemble light microscopic observation of native tissues in Fig. 2D. The flakes are slightly curved and they display an edged interface with the cytoplasm (see arrowheads), and a smooth interface between alternating electron-dense and electron-lucent striations. The surrounding cytoplasm is densely filled with glycogen rosettes (asterisks). Essentially, no glycogen or other cytoplasmic content is incorporated in the electron-lucent material of the putative flakes. Bars: (A), 2 µm; (B) and (C), 1 µm.

TEM observation of samples preserved by conventional chemical fixation, dehydration and resin embedding at room temperature undoubtedly identified sources of the reflective elements described above in the form of numerous rhomboid inclusions inside the epidermis of the ovisac. The rhomboids were preferentially oriented in parallel to the ovisac surface (Fig. 4A). Their occurrence was restricted to the inside of the epithelial cell layer, and their abundance was consistent with light microscopic observations. However, comparison with CRM data also highlighted a discrepancy: the flake-like nature of the reflective entities could not be confirmed. Although individual electron-dense rhomboids congregated in a parallel orientation as groups, they did not show any physical linkage between each other. The empty space between neighboring rhomboids coincided with the electron-lucent overall appearance of the epithelial cell cytoplasm. Suspecting that these ‘empty spaces’ were due to the harsh effects of organic solvents on sample preparation at temperatures above zero, we investigated whether cryotechniques in preparation of samples for TEM could better maintain the flake-like nature of the cellular inclusions indicated by CRM.

### TEM preparation at cryo-temperature preserves mesostructured, flake-like inclusions

For comparison with conventional embedding, we processed instantly high-pressure frozen ovisacs by freeze substitution (FS). The FS protocol, aiming at dehydration and fixation at a very low temperature, was kept particularly short by agitation of the samples in a commercial FS unit (Goldammer et al., 2016). As shown in Figs. 4B and 4C, cryopreparation had a profound influence on the improved preservation of the cellular inclusions we described in this study. The epidermal cell detail at low magnification (Fig. 4B) displays a cryopreparation with well-preserved cytoplasmic content, including many ribosomes and glycogen. The cuticle is covered with a continuous, brush-like surface layer, which is not preserved by conventional chemical processing. Moreover, it is folded, in accordance with the domain-like imaging of ovisac lining by CRM (Fig. 2D) and polarization microscopy (Fig. S1) on native samples. Rhomboids, most of them aligned in groups, are separated by electron-lucent areas that are confined against areas of ribosome and glycogen-containing cytoplasm. Visualization of longitudinally cut inclusions at higher magnification (Fig. 4C) indicates that the electron-dense rhomboids and the electron-lucent material are combined as integral parts of the flake-like structures, which resemble the cellular inclusions observed by light microscopy in native tissues of Fig. 2D. Consequently, our cryotechnical approach maintained the integrity of an alternating material composition. The flake-like characteristics of the cellular inclusions became clear, if their ‘zebra-striped’ patterns were analyzed in detail: the interface between electron-dense and electron-lucent stripes appears to be blurred as a result of tangential sectioning, while most of the outer edges, and especially those running in perpendicular to striations, are visualized as sharp lines. Taken together with the results by CRM, the three-dimensional consequence of these observations in the TEM is that the mesostructured cellular inclusions represent striated flakes, which (in terms of exemplification) resemble the geometry of ‘crinkle-cut potato chips’. Given the apparent superiority of the cryopreparation samples, we performed all subsequent studies (observations in different *A. franciscana* strains, electron tomography, EDX spectra analysis, and electron diffraction) on basis of these methods.

### Initial evidence for participation of crystals in mesostructured cellular inclusions

A central point to our findings is whether the two striated components of the inclusions in the ovisac epithelium are of monocrystalline, polycrystalline, paracrystalline or amorphous nature, inclusively of the possibility that alternating assembly of crystalline and amorphous constituents might be necessary for formation of the ‘zebra stripe’ patterns.

The rhomboid shape of the electron-dense component and its sharp-lined interface are arguments for their crystalline origin. However, routine TEM images at high-magnification, recorded at 120 kV, did not display any evidence of grid-patterns of monolithic crystals- irrespective of sample preparation method; whether chemically fixed samples, processed at room temperature, or high-pressure frozen, freeze-substituted samples were used. To raise the resolution for TEM imaging of the HP frozen, freeze-substituted samples we (i) observed samples at 200 kV, and (ii) performed electron tomography at 200 kV to generate images from virtual sections, more than an order of magnitude thinner than sections cut with an ultramicrotome.

**Figure 5:**
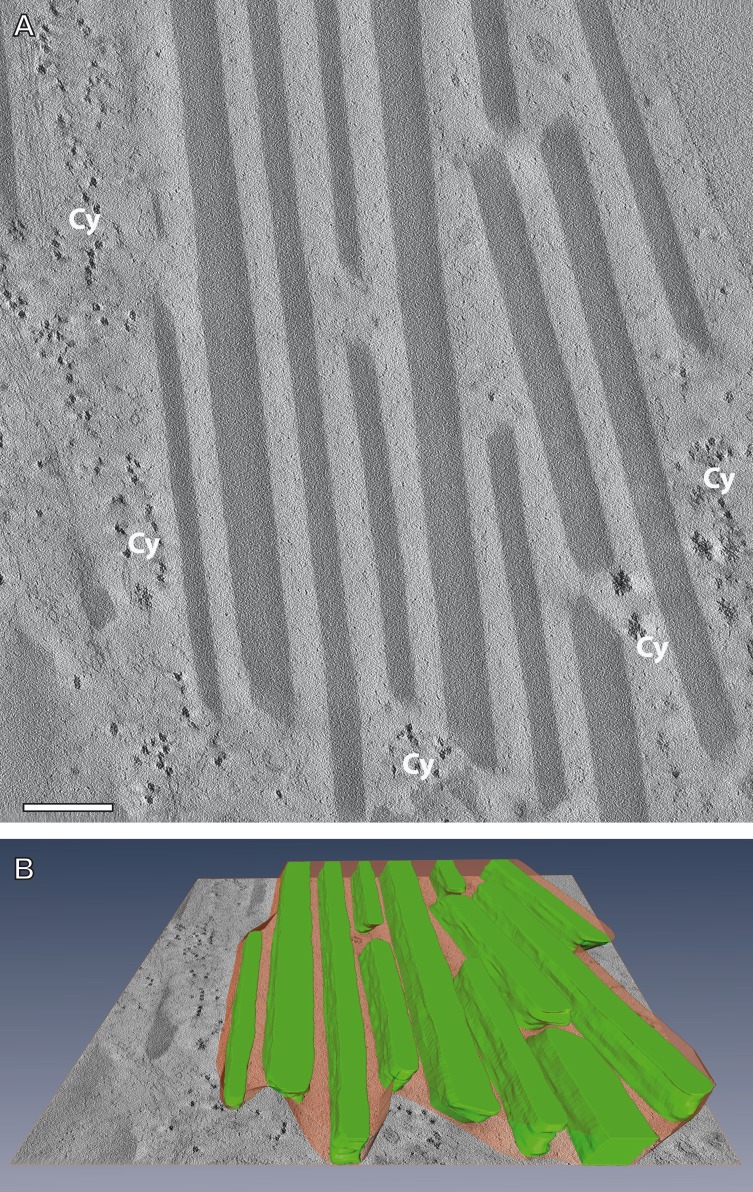
Electron tomography of a mesostructured flake-like inclusion in the cytoplasm of *A. franciscana*. (A) Individual virtual section of a slice thickness of ca. 0.5 nm generated from a 3D-reconstructed tilt series. The sectioned flake contains alternating electron-opaque and electron-lucent striations. Note a faint contrast between the cytoplasm (Cy) and the more electron-lucent material of the striations. (B) 3D reconstruction of the flake from virtual sections indicates the incorporation of the electron-lucent material between rhomboid electron-dense materials. Bar in (A) 200 nm.

Neither the high resolution analysis of ultrathin sections at 200 kV (Fig. S2), nor studies of virtual sections obtained by reconstruction of electron tomographic tilt series (Fig. 5) could resolve grid-like patterns to give evidence of a monocrystalline nature of the mesostructured inclusions. Instead, they displayed contrasting modulations within the stripe patterns, conceivable to be caused by small, densely packed polycrystals. Besides, the interface between the electron-lucent stripes and the cytoplasm could be identified on the basis of a noticeable contrast in virtual tomographic sections. Accordingly, the interface border follows a concisely continuous contour defined by the sharp outer edges of the electron-dense material. The formation of such mesostructured flakes requires coordinated assembly of two cellular material components under participation of crystal growth; it cannot be explained simply by aggregation processes of proteinaceous material, as it would happen during the formation of irregularly shaped inclusion bodies (Fink, 1998).

**Figure 6:**
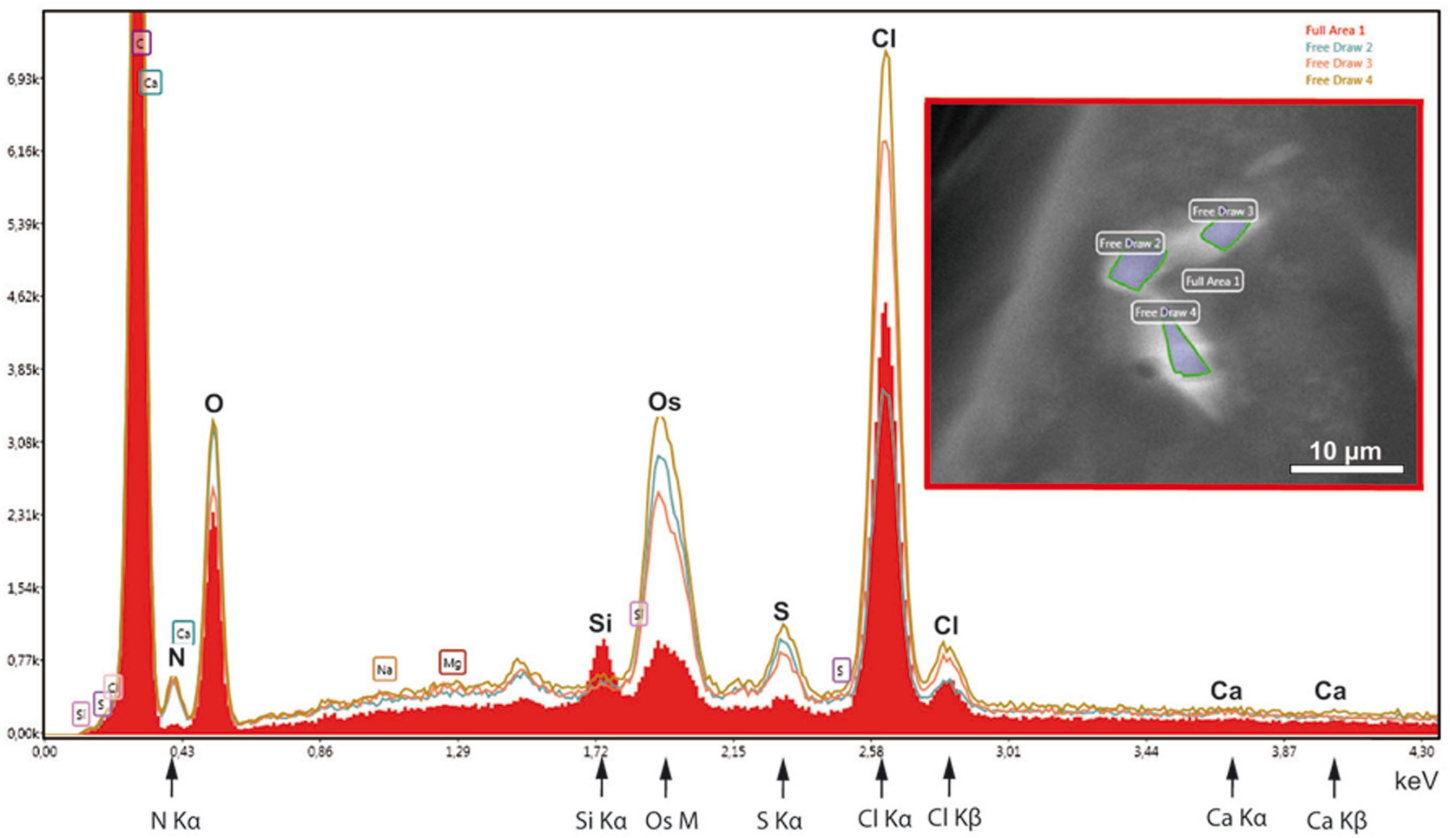
EDX-analysis of the element composition within cellular inclusions. The spectra of encircled crystal-like inclusions (free draw 2–4) were compared with the spectrum of the overall area (underlaid red). The K_*α*_-peaks of nitrogen are all significantly higher in the free-draw regions. They are not affected by Ca- L_*α*_-radiation, since calcium, a major candidate for inorganic crystallization, is not enriched in the free-draw regions; there are no K_*α*_- and K_*β*_ peaks present at the marked positions of the spectra. The enrichment in nitrogen, possibly caused by a protein concentration higher than in the surrounding cytoplasm, coincides with the enrichment in sulfur indicated by S-K_*α*∕*β*_ peaks. The chlorine peaks, in contrast, display variations in their intensity. Note also that a Si K_*α*_-peak is generated within the overall area, because of the excitation of Si wafer material underneath the section (see ‘Material and Methods’). Moreover, the crystal-like inclusions (free-draw 2–4) show enrichment in osmium that is indicated by more prominent M-peaks in comparison with the overall area. Contrasting with osmium is the precondition for visibility of the cellular inclusions in the electron backscattering mode of the scanning electron microscope, notably, without resolving their characteristic striated patterns (boxed insert).

In order to test the supposed crystalline nature of the cellular inclusions more in detail, we applied selected area electron diffraction (SAED) in a 200 kV- electron microscope to cellular inclusions in TEM thin sections of HP frozen, freeze-substituted samples. The marked regions encircling the inclusions did not generate spot- or ring SAED patterns in evidence of a mono- or polycrystalline nature, respectively (Fig. S3). This outcome contrasts with data of other superstructured biocrystals formed during mineralization; the so-called mesocrystals display SAED patterns of their inorganic crystalline components, regularly (Bergström et al., 2015). Therefore, we wanted to know whether elements typically involved in mineralization are absent in the striped flakes of the ovisac lining. We applied energy dispersive X-ray analysis (EDX) in the SEM to semithin resin sections (Fig. 6), with the result that elements characteristically involved in the formation of mineralization, and in particular calcium and iron were not detected in significant amounts within the flakes. Phosphor was not detectable at low amounts, because the measurement of its K_α_-peak was obscured by the presence of the osmium required for contrasting of the cellular inclusions.

Instead of elements typically involved in biominerialization, we found significantly higher amounts of nitrogen in marked regions of the flakes, if compared with the overall cytoplasm (Fig. 6). Since nitrogen participates in peptide bonds of amino acids of proteins, this result may indicate a higher concentration of proteins within the flakes, than the protein concentration of the surroundings. Such higher package density of proteins could possibly be achieved by crystallization.

**Figure 7:**
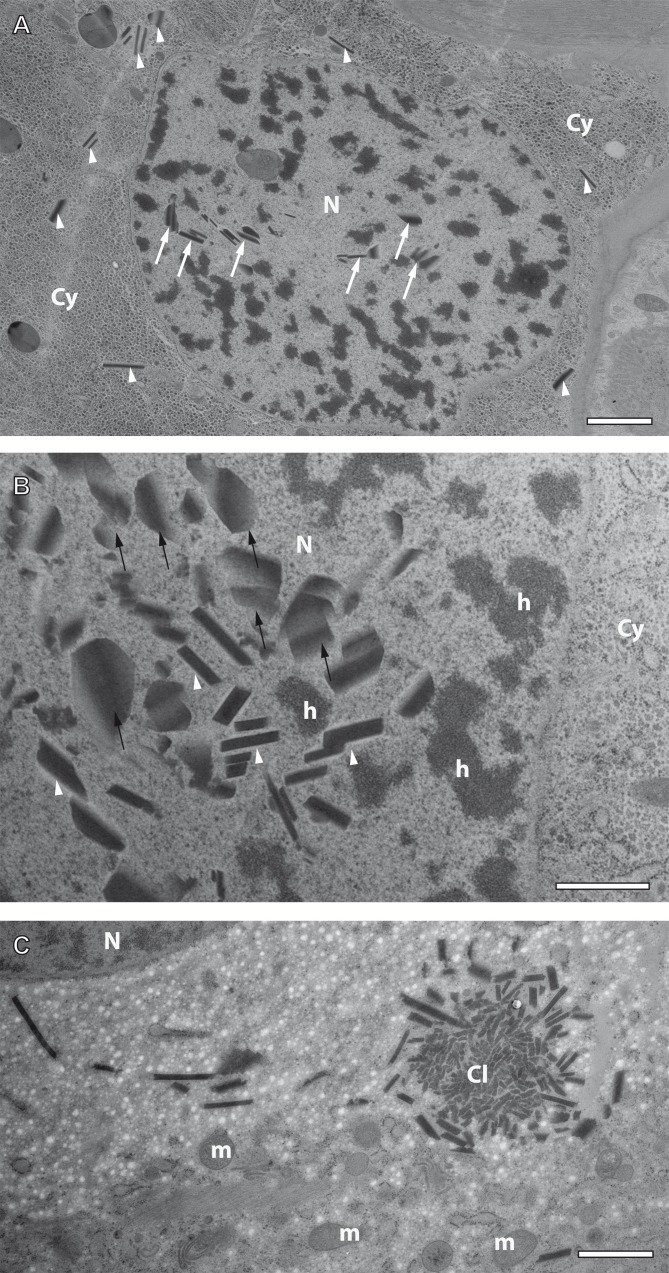
Preliminary ultrastructural evidence for the formation of mesostructured inclusions inside both the cytoplasm and nucleoplasm of *A. franciscana* obtained by cryopreparation. (A) Epidermal cell displaying small inclusion at low abundance both in its cytoplasm (arrowheads) and its nucleus (arrows). (B) Detail displaying inclusions within a nucleus showing small electron-dense rhomboids with an electron-lucent coat (arrowheads) and small, flake-like inclusions in their neighborhood (arrows). (C) Cytoplasmic cluster (Cl) of small, putative precursors surrounded by small rhomboids similar to those observed in the cell nucleus in (B). The cytoplasm of the cell in (C) contains areas with numerous electron-lucent spots. Given the rarity of the observation it could not be decided whether these spots are related to the assembly process, or not. Artifact by ice crystals might have caused or at least influenced them. N- nucleus, Cy- cytoplasm, h- heterochromatin, m- mitochondria. Bar in (A) 2 µm. Bars in (B) and (C) 1 µm.

### Formation of crystal-like superstructures comprises both the cytoplasm and the nucleoplasm

Crystal-like superstructures are omnipresent in the cytoplasm of the epithelial layer of the ovisac lining. Figure 4 displays them at high abundance in the cytoplasm of the epithelial epidermis with preferential orientation in parallel to the cuticle. They represent also regular constituents of epithelial cell nuclei, although the likelihood for finding them inside thin sections is reduced. Figure 7A displays a glycogen-rich epithelial cell containing both cytoplasmic and nuclear inclusions, seemingly at an early stage of their development; small, and sometimes individual rhomboid crystals in both the nucleoplasm and the cytoplasm, are displayed at low abundance. Observations in the nucleoplasm of an epidermal cell at higher magnification, revealed that most small, individual rhomboids located next to already assembled flakes possess an electron-lucent ‘coat’ at their outside (Fig. 7B). On rare occasions, we also observed an uncoated cluster in the cytoplasm (Fig. 7C), which could give rise to a clue about very early stages of the formation of individual rhomboids. The figure displays putative small electron-dense precursors surrounded by small sharply edged rhomboids. Whether the abundant presence of white spots in the surrounding is related to the ‘coating’ of individual rhomboids, or whether they represent artifacts caused by ice crystals cannot be decided from this by chance-observation.

## Discussion

### Evidence for the participation of crystallization in the formation of mesostructured cellular inclusions

TEM revealed cellular inclusions in the epithelial cell layer of the ovisac lining of *A. franciscana*. As demonstrated by electron tomography, these inclusions form flakes with striped patterns aligned exactly in parallel and edged at the interface to the cytoplasm and also at the interface between the electron-dense and the electron-lucent striations. From our point of view, the assembly of such patterned flakes is conceivable only along crystalline growth frontiers. The resulting ‘zebra stripes’ are of a large scale compared with the elementary constituents of the material, i.e., atoms and molecules. In consequence, the striated flakes resemble ‘mesocrystals’—a term coined by Cölfen for a new class of solid materials (Cölfen & Antonietti, 2005). Mesocrystals are assembled by a particle-mediated process that links crystallographically oriented inorganic nanocrystals with interspacing amorphous organic or inorganic substances. The nanocrystals are thought to undergo a mesoscale-oriented self-assembly because of preferential surface interactions with the interspacing substance. Mesocrystals are formed naturally during biomineralization, e.g., in bones and mollusk shells, where they result in light weight composites with high strength and fracture toughness. The combination of crystallinity with porosity might also result in mesocrystals with extraordinary properties for sensing, photovoltaic and photocatalytic devices based on a faster, more efficient electron transfer (Zhou & O’Brien, 2008; Bergström et al., 2015). The mesostructures found in the ovisac lining of *A. franciscana*, did not contain substantial quantities of elements potentially involved in inorganic crystallization, nor did they display electron diffraction patterns. Therefore, and because of the increased nitrogen content inside the inclusions, we suggest the assembly of proteinaceous (poly)crystalline subunits in striated superstructures, under participation of yet unknown non-proteinaceous biopolymers. It would apply to a concept of nonclassical assembly of organic particles into building units, which may favor stacking of the flakes by a decrease in surface energy (Niederberger & Cölfen, 2006; Ma, Cölfen & Antonietti, 2006). Our observation of small individual rhomboids covered with a layer of electron-lucent material, indicates similarities with a hypothetic scenario suggested for mesocrystal formation based on inorganic crystalline subunits, since it requires coating of the crystalline subunits with an adherent prior to assembly into mesostructured arrays (Niederberger & Cölfen, 2006; Zhou & O’Brien, 2008). More detailed studies of the assembly of the striated flakes in the lining of ovisacs would require their isolation and enrichment for application of X-ray diffraction techniques and mass spectroscopy.

### Preservation of mesostructures by application of cryotechniques

Numerous crystal-like cytoplasmic and nuclear inclusions have been previously observed in TEM (for review: Fawcett, 1981; Ghadially, 1997). They have usually been assumed to be of proteinaceous nature, but the specific proteins have rarely been identified (Fawcett, 1981). The question of how crystals, many times larger than nuclear pore complexes (for review on NPCs see Suntharalingam & Wente, 2003), could assemble inside the nucleus has been addressed by Gouranton & Thomas (1974) by studying large intranuclear crystals in midgut cells of the whirliglig beetle *Gyrinus marinus*. Their autoradiographic experiments provided evidence for the synthesis of crystal proteins in the cytoplasm. Consequently, these proteins had to enter the nuclei via NPCs for assembly into nuclear crystals. From our point of view, such a scenario is conceivable with the simultaneous generation of inclusions both in the cyto-and nucleoplasm of the epidermal cells of *A. franciscana*.

Comparison of our data obtained by cryopreparation with samples that were chemically processed at room temperature clearly showed that the later are strongly affected by extraction of cellular material, such as proteins and carbohydrates. Besides a general elution, the extraction acts specifically on the electron-lucent content of the mesostructures. In consequence, the flake-like nature of these inclusions cannot be preserved by conventional ways. The ‘remains’ in the form of electron-dense rhomboids are reminiscent of numerous similar observations, made previously in conventionally processed tissues, which have been interpreted as crystalline inclusions (e.g., in the liver of the salamander *Batrachoseps* ( Hamilton, Fawcett & Christensen, 1966); in equine Schwann cells (Force, Jortner & Scarrat, 1986); in endometrial luminal epithelium and trophectoderm of ovine uteroplacental tissues (Gray et al., 2005); in tubular kidney epithelial cells of patients with the Fanconi syndrome, dysproteinemia-related nephropathy, or with kidney transplant (Stokes et al., 2006; Kim et al., 2014; Lum, Higgins & Tan, 2014). Histological studies of Reinke crystals in Leydig tumor cells made aware that the crystals themselves might be prone to degradation/dissolution. Mesa et al. (2015) reported that Reinke crystals dissolve rapidly in aqueous solutions (10% formalin) and very slowly and incompletely in alcohol, and they stressed practical consequences of their finding for the histopathologic tumor diagnosis.

The work by Gray et al. (2005) contains a hint for the kind of losses that we may face in consequence of conventional sample preparation: Gray et al. applied immunogold labeling for galectin-15, a *β*-galactoside-binding lectin, to TEM sections of ovine uteroplacental tissues and found an intense immunolabeling to rhomboid crystals, confined within otherwise heavily extracted cells. This preparation, therefore, could have induced a loss of structure-related sugars, as natural binding partners to lectin proteins. In our opinion, losses of sugars and polycarbohydrates should be of general concern for studies of protein aggregations and crystallization in cells and tissues. We recommend HPF in combination with accelerated FS under agitation as a means to prevent losses of polycarbohydrates. Advantages in sample preparations have been demonstrated most recently in a different context, namely for preservation of algal starch (Goldammer et al., 2016). Here, cryopreparation revealed the true nature of cellular inclusions in the ovisac lining of *A. franciscana*, for our point of view, most likely by prevention of losses of electron-lucent polycarbohydrates.

### Reflective, light dispersing properties realized by mesoscopic superstructures

Confocal Reflection Microscopy (CRM) without any chemical sample processing has been proven as the method of choice for identification of mesostructured cellular inclusions at the ovisac lining of *A. franciscana*, but it could only provide an incomplete impression of the coverage of the ovisac with these structures, due to folding exceeding the dimension of the *z*-stack. As shown in Figs. 2D and 3, the flake-like inclusions are not strictly oriented; they present variability in the incidence angles to incoming light. CMR revealed the optical active properties of the flake-like inclusions resulting from dispersion of visible light by grid-like striations. It also demonstrated variability of the reflection properties of individual flakes, which can be explained by differences in the orientation inside cells, variation in number and distance of the striation, and curvatures of the flakes. We conclude that the mesoscopic structure of the flakes is optimized for the reflective dispersion of yet unquantified portions of visible incident light that falls onto the ovisac surface.

If compared with other photonic structures in biology, and especially with those in aquatic organisms (for review:  Vukusic &Sambles, 2003; Gur et al., 2017), the optical active flakes of *A. franciscana* differ significantly from previously reported photonic crystals. The crystalline guanine plates of fish scales, for instance, are not curved but planar; they generate iridescent colors on the basis of their surface-equidistant arrangement as tunable multilayers (Fudouzi, 2011). Alternating layers of exactly hexagonal-shaped guanine crystals are responsible for the spectacular colors of male sapphirinid copepods (Gur et al., 2015). Very much in contrast to these examples, every individual flake-like inclusion in *A. franciscana* generates iridescence by virtue of its own striated superstructure.

### On the relation between light and reproduction of *A. franciscana*

As early as 1973, Sorgeloos described a triggering effect of light on the hatching mechanism of encysted *A. franciscana* during the course of oviparous reproduction. A similar phenomenon of a light-activated hatching has been reported more recently for *A. urmiana* (Asil et al., 2012). According to Van der Linden et al. (1989) and Van der Linden et al. (1991) light receptors inside the encysted embryo, perhaps consisting of porphyrin or haempigments, are responsible for activation of hatching response at wavelengths in the blue and green spectral region (action peaks: 450–470 nm and 525–575 nm). If rehydrated embryos at a stage of diapause can be stimulated via light receptor signaling, one may wonder what effect a similar light stimulus would have on eggs that are still inside the ovisac for completion of their development. In our point of view, it appears necessary to identify the developmental stage where photoreception begins. If the activation of such light receptors happens prior to the release of the cysts in *A. franciscana* and perhaps also in other species, there would be good reason to suppress preterm hatching within the ovisac by spanning a light-reflecting ‘umbrella’. The fact that mesostructures are also located inside cell nuclei could be interpreted as a detail in favor of the effectiveness of the ‘umbrella’, since nuclei devoid of mesostructures, incorporated in a very flat epidermal cell layer, would act as holes in the ‘umbrella’s’ screen.

From our point of view, an ‘umbrella’ would be required until the multilayered cyst shell consisting of a non-cellular chorion layer and embryonic cuticle has developed for protection against multiple factors such as light or mechanical and thermal stress. Completion of the protective shell includes the assembly of a chitin-containing layer under participation of chitin-binding proteins (Liu et al., 2009; Ma et al., 2013). The fibrous architecture of this layer of the embryonic cuticle resembles the typical arrangement of chitin in the epidermal cuticle of arthropods such as crustaceans. It consists of horizontally parallel and vertically twisted chitin microfibrils (Havemann et al., 2008). Most recently Wilts et al. (2017) reported that chitin polymers can also assemble quite differently, in the form of biophotonically active gyroid crystals located in iridescent wings of butterflies. It appears that these gyroids act as an alternative to optical active guanine crystals, which are used in nature for the manipulation of light (Gur et al., 2017). Notably, neither the gyroids made of chitin nor guanine crystals show any structural similarity to the mesostructured flakes assembled in the epidermal cells of the ovisac of *A. franciscana*.

Another aspect of the relation between light and reproduction concerns the influence of photoperiod on the reproduction mode. Nambu, Tanaka & Nambu (2004) found that the ratio between oviparity and ovoviviparity in cultured *A. franciscana* is greatly affected by the light-dark cycle, and much less by temperature. We speculate that this reflective ‘umbrella’ could act as threshold barrier; accordingly, the predominant release of live larvae would require sufficient light exposure for a certain period of time. However, one has also to consider the finding by Dai et al. (2011) that predominantly diapause- and nauplii-destined oocytes of *A. parthenogenetica*, reared by application of differential light exposure, expressed genes already differentially at this early developmental stage. Threshold conditions for light exposure at a later stage of embryogenesis, therefore, might serve as final check of the environmental factor ‘light’.

## Conclusions

The results of this study may have implications for optimization of light-dark cycles in rearing *Artemia* culture as feed for fish farming. In addition, we suggest that the *Artemia* ovisac studies could be extended and developed in future as a new model system for investigations into the physics and biochemistry of mesostructural formation.

##  Supplemental Information

10.7717/peerj.3923/supp-1 Figure S1 Ovisac of *A. franciscana* visualized by using polarized light microscopy (A) overview, with the spine (asterisk) and central surface region in focus. (B) Regions covered with flake-like structures, separated from each other through clefts. (C) Detail. An arrow points towards flakes with faint striations, which resemble the mesostructures resolved by CRM in Fig. 3. Bar, 100 µm. (B) 50 µm. (C) Bar, 20 µm.

10.7717/peerj.3923/supp-2 Figure S2 Selected Area Electron Diffraction (SAED) of cytoplasmic inclusion of * A.franciscana* (A) Bright field image with encircled areas of diffraction 1 and 2. (B) Diffraction of the bare epoxy resin. (C and D) The diffraction images of areas 1 and 2 both are devoid of patterns in evidence of a polycrystalline or monocrystalline nature of the embedded material.

10.7717/peerj.3923/supp-3 Figure S3 High resolution transmission electron microscopy of the transition zone between electron lucent and electron-dense components of the cellular inclusion in *A. franciscana* at 200 kV Note a diffuse change in contrast in the upper third of the micrograph and granular fine structures of the assembled electron-lucent and electron-dense material on both sides.

10.7717/peerj.3923/supp-4 Movie S1 Ovisac of female *A. franciscana* at a stage of initial vitellogenesis of oocytes in the oviduct The ovisac still does not contain eggs.

10.7717/peerj.3923/supp-5 Movie S2 Ovisac of female *A. franciscana* containing the first couple of eggs At this stage glittering flakes become apparent at the ovisac surface. (The copulating male was separated from the female to provide an undisturbed view at the ovisac).

10.7717/peerj.3923/supp-6 Movie S3 Male enclosing a female *A. franciscana* with its clasping organs At this time point the ovisac still does not contain eggs that could be fertilized.

## Additional Information and Declarations

### Data Availability

The following information was supplied regarding data availability: Figshare: https://figshare.com/s/1519e08f58da6366a274.
