# Supplementary figures and images for: Novel mesostructured inclusions in the epidermal lining of Artemia franciscana ovisacs show optical activity
Source: PeerJ. 2017 Oct 27;5:e3923. doi: 10.7717/peerj.3923 (PMC5661469; doi:10.7717/peerj.3923)

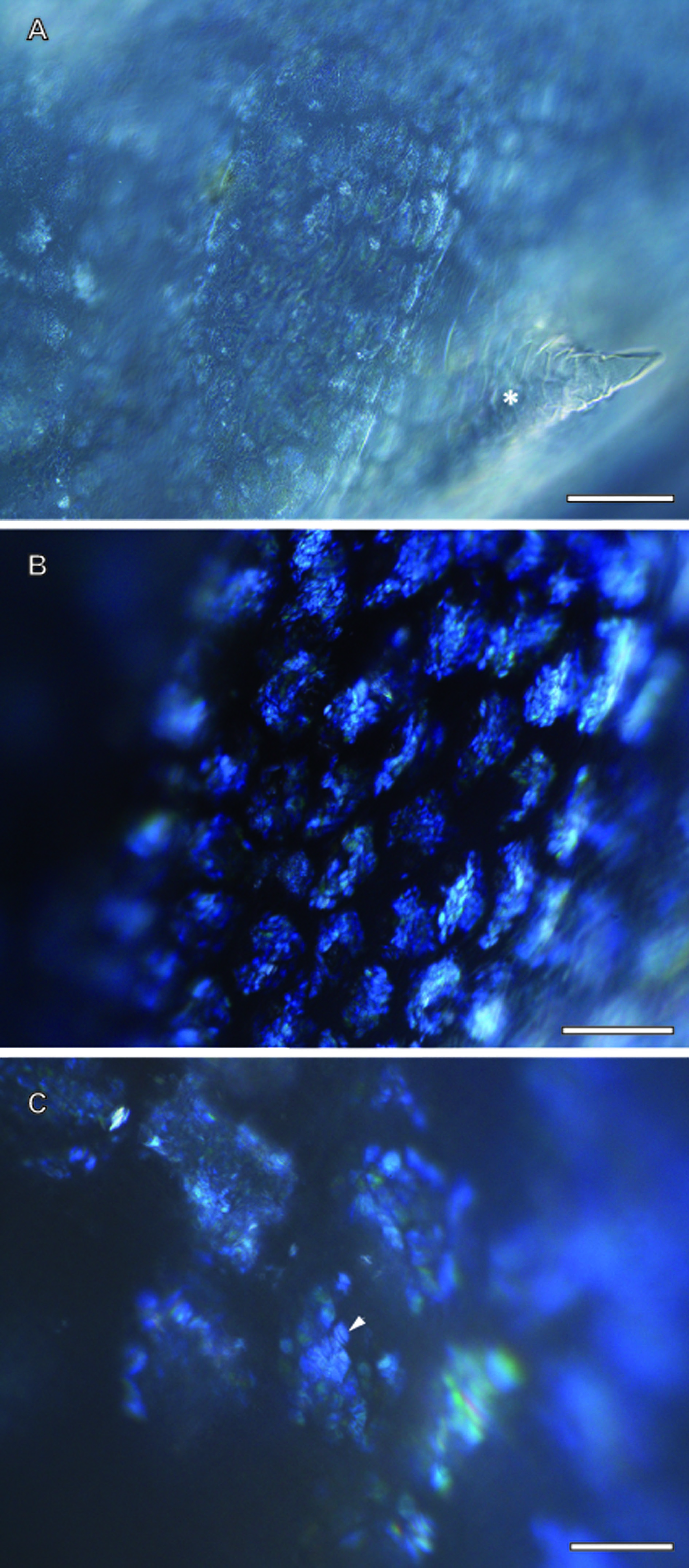

Supplement: Figure S1 — (A) overview, with the spine (asterisk) and central surface region in focus. (B) Regions covered with flake-like structures, separated from each other through clefts. (C) Detail. An arrow points towards flakes with faint striations, which resemble the mesostructures resolved by CRM in Fig. 3. Bar, 100 µm. (B) 50 µm. (C) Bar, 20 µm. [file peerj-05-3923-s001.jpg]

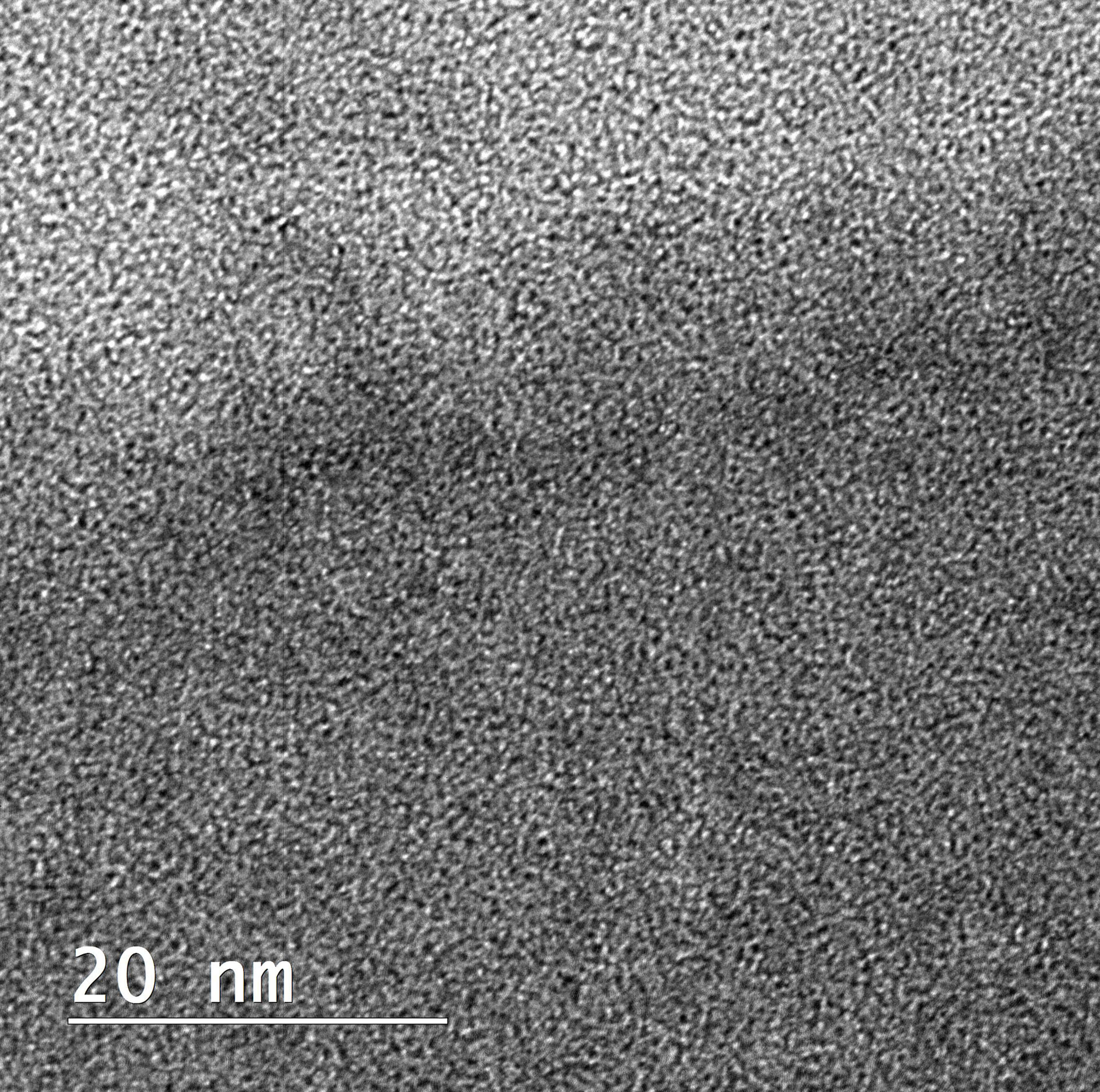

Supplement: Figure S2 — (A) Bright field image with encircled areas of diffraction 1 and 2. (B) Diffraction of the bare epoxy resin. (C and D) The diffraction images of areas 1 and 2 both are devoid of patterns in evidence of a polycrystalline or monocrystalline nature of the embedded material. [file peerj-05-3923-s002.jpg]

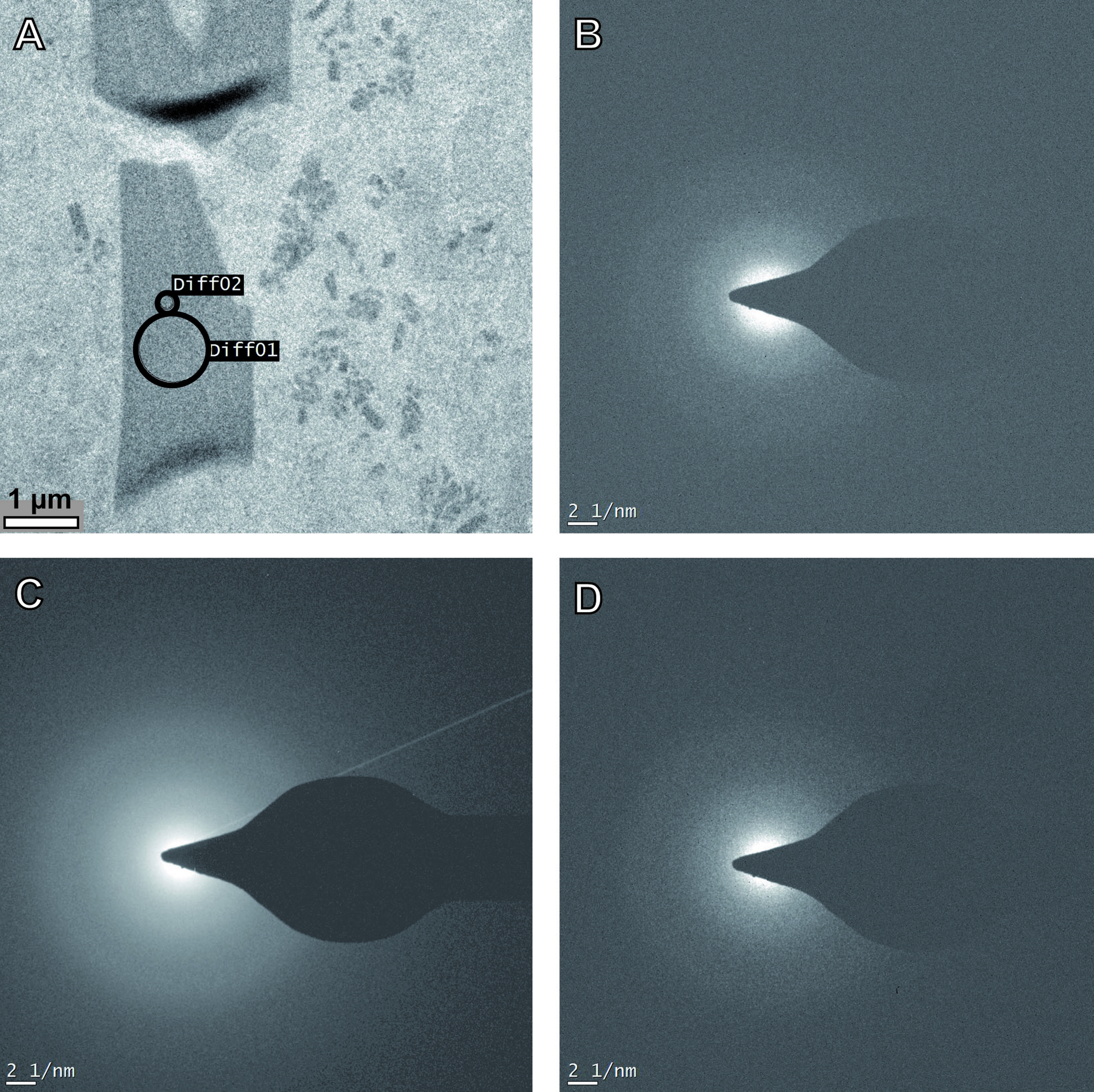

Supplement: Figure S3 — Note a diffuse change in contrast in the upper third of the micrograph and granular fine structures of the assembled electron-lucent and electron-dense material on both sides. [file peerj-05-3923-s003.jpg]
